# Stereotactic Ablative Radiation Therapy for Metastatic Renal Cell Carcinoma – A Review of Evidence

**DOI:** 10.15388/Amed.2025.32.1.18

**Published:** 2025-02-18

**Authors:** Hima Bora, Gautam Sarma, Partha Pratim Medhi

**Affiliations:** 1 Department of Radiation Oncology, Tezpur Medical College and Hospital, Tezpur, Assam, India; 2 Department of Radiation Oncology, All India Institute of Medical Sciences, Guwahati, Assam, India

**Keywords:** stereotactic ablative radiation therapy, metastatic renal cell carcinoma, *oligometastatic* renal cell carcinoma, oligoprogressive renal cell carcinoma, renal cell carcinoma, stereotaksinė abliacinė spindulinė terapija, metastazavusi inkstų ląstelių karcinoma, oligometastazavusi inkstų ląstelių karcinoma, oligoprogresuojanti inkstų ląstelių karcinoma, inkstų ląstelių karcinoma

## Abstract

Because of its remarkable precision in providing targeted radiation, recent evidence supports *Stereotactic Ablative Radiation Therapy* (SABR) as a promising non-invasive treatment approach for metastatic renal cell carcinoma, minimizing harm to adjacent healthy tissues. With regards to its heterogeneous nature with diverse clinical presentations, rapid progression and metastatic potential, *Renal Cell Carcinoma* (RCC) is known to make therapy more challenging, and also to reduce the survival rates. Even though *Immune Checkpoint Inhibitors* (ICIs) remain the gold standard for treating metastatic RCC (mRCC), certain patients with one or a few distant metastases seem to have a longer survival period if the metastases are surgically removed. However, complete responses are not always the case, with radiation being increasingly incorporated as a component of multidisciplinary care. Moreover, studies proving the immunogenic qualities of hypofractionated SABR and the safety and potential of combining SABR with immune-based and surgical therapy for mRCC are becoming more prevalent in the literature. SABR helps induce local inflammation with the tumour, promoting T cell activation and antigen presentation. Multiple retrospective and prospective reports have also demonstrated that SABR assigned to the metastatic locations of mRCC, while using ablative dosages, achieves high local control rates with a good toxicity profile, thus disproving earlier theories of RCC radioresistance. This review outlines the key evidence favouring SABR being administered to metastatic tumours, including the results of recent prospective phase 2 trials in patients with oligometastatic, oligoprogressive, and unselected mRCC. The body of data that has been gathered points to SABR as a promising indicator that is being utilized more and more in the multidisciplinary management of mRCC.

## Abstract (it)

Naujausi tyrimo duomenys patvirtina, kad stereotaksinė abliacinė spindulinė terapija (SABR) yra perspektyvus neinvazinis metastazavusios inkstų ląstelių karcinomos gydymo būdas, nes ji pasižymi nepaprastu tikslumu ir kuo mažiau pažeidžia gretimus sveikus audinius. Yra žinoma, kad inkstų ląstelių karcinoma (RCC) dėl savo heterogeniškumo, klinikinių požymių įvairovės, greito progresavimo ir metastazavimo potencialo apsunkina gydymą ir mažina pacientų išgyvenamumo rodiklius. Nors imuninių kontrolinių taškų inhibitoriai (ICI) tebėra metastazavusios RCC (mRCC) gydymo aukso standartas, kai kurių pacientų, turinčių vieną ar kelias tolimas metastazes, išgyvenamumas pailgėja, jei metastazės pašalinamos chirurginiu būdu. Tačiau ne visada pavyksta pasiekti visišką atsaką, todėl vis dažniau į daugiadisciplininę priežiūrą įtraukiama spindulinė terapija. Be to, literatūroje vis dažniau pasirodo tyrimų, įrodančių hipofrakcionuotos SABR imunogenines savybes ir SABR derinimo su imuniniu ir chirurginiu mRCC gydymu saugumą ir galimybes. SABR padeda sukelti vietinį uždegimą su naviku, skatina T ląstelių aktyvaciją ir antigeno pateikimą. Daugybė retrospektyvinių ir prospektyvinių tyrimų taip pat parodė, kad, taikant SABR, skirtą metastazavusiam mRCC, naudojant abliacines dozes, pasiekiamas aukštas vietinės kontrolės lygis ir geras toksiškumo profilis, o tai paneigia ankstesnes teorijas apie RCC radiorezistentiškumą. Šioje apžvalgoje įvertinami pagrindiniai įrodymai, patvirtinantys, kad SABR skiriama metastazavusiems navikams, įskaitant neseniai atliktų prospektyvių 2 fazės tyrimų su pacientais, sergančiais oligometastazavusiu, oligoprogresuojančiu ir neatrinktu mRCC, rezultatus. Surinktų duomenų visuma rodo, kad SABR yra perspektyvus rodiklis, kuris vis dažniau naudojamas taikant daugiadisciplininį mRCC gydymą.

## Introduction

90% of kidney cancers are renal cell carcinomas (RCCs), which are a heterogeneous group of diseases with a wide range of clinical courses and rapid progression. The mode of progression set forth as per *Response Assessment Criteria in Solid Tumors* (RECIST) guidelines [1,2]. RCCs can be diagnosed as having either an upfront metastatic disease (synchronous) or metastasis after local treatment (metachronous), with a 20% and 25% incidence rate, respectively [3]. *Immune Checkpoint Inhibitors* (ICIs), *Tyrosine Kinase Inhibitors* (TKIs), or a combination of the two is the standard of care for metastatic RCC (mRCC), and it has broadened treatment options and increased the survival [4–8]. Nephrectomy and surgical resection of the metastases appear to prolong the survival in a subset of patients with one or a few distant metastases [9,10]. Nevertheless, complete responses are not always achieved, making radiation a crucial component of multidisciplinary care, particularly in cases where surgery may not be the optimal approach. Its usage has also gained support in recent international practice [11]. Because of the enduring belief that RCC is biologically radioresistant, radiotherapy has not been applied extensively. Reportedly, isolated RCC cells *in vitro* were among the cell types most radioresistant to the standard dosages of the radiation therapy, according to early preclinical research [12], which solidified this opinion. However, radiation therapy for RCC has become more widely used since the development of contemporary dose-escalated radiation employing *Stereotactic Ablative Radiation Therapy* (SABR) [13–16]. There have only been retrospective reports found thus far among the recent systematic reviews for both SABR [17] and metastasectomy [18] in advanced RCC.

Clinical studies of SABR in conjunction with surgery or systemic therapy for patients with metastatic disease are being conducted as a result of growing evidence suggesting that the radiation therapy has immunomodulatory effects. The following studies [19–23] provide evidence of prior experience with stereotactic high-dose fraction radiation therapy, which has a high *Local Control* (LC) rate for various tumour types, including both primary and metastases in RCC patients. In this review, we will describe the most important recent evidence from prospective phase 2 studies so far, as well as areas of ongoing research for integrating SABR in the treatment of mRCC.

## Review of Literature

Search Strategy: Relevant prospective clinical trials identified by conducting a comprehensive literature search related to SABR in mRCC. After a systemic search using the following keywords: kidney neoplasms, kidney cancer, kidney tumour, renal cell carcinoma radiosurgery, radiosurgery, SABR, stereotactic, metastasis, and oligometastasis, oligoprogression (conducted in *Pubmed, Google Scholar, Scopus*) prospective studies with restriction to phase 2 clinical trials till date have been included. All of them were published in the English language. The below-mentioned studies matched the criteria considered valuable for this crucial review (Table 1).

**Table 1:** Phase 2 trials using SABR in metastatic renal cell carcinoma

Study design	Treatment	Sample	Follow-up (months)	Outcomes	Toxicities	Interpretation
***SABR for Oligometastatic RCC in Combination With Systemic Therapy***						
Hannan et al. (2021), single-arm, mRCC (≤6 lesions) [25]	One fraction (total dose 21–27 Gy) or three fractions (total dose 26.5–33 Gy) SABR to the metastatic sites followed by HD IL2. All patients had a prior nephrectomy	30	24	By RECIST, overall response rate was 16% (4/25 patients). Median OS = 37 months, Median PFS = 2 months	Grade ≥3 AEs=22, no grade 5AEs	SABR failed to enhance the HDIL2 response rate
Siva et al. (2022), single-arm, mRCC (≤5 lesions) [26]	One fraction (total dose 20 Gy) or ten fractions (total dose 30 Gy) SABR followed by Pembrolizumab 8 cycles.	31	28	2 yrOS=74% 2yr PFS=45%	13% grade 3 AEs	SABR and Pembrolizumab short-course are well-tolerated and have good LC
***SABR in Combination with Systemic Therapy for Unselected mRCC***						
Masini et al. (2022). single-arm [28]	1^st^ infusion of Nivolumab followed by 30 Gy in three fraction SABR	69	26	ORR=17% DCR= 55%. Median PFS =5.6 months, median OS=20 months	grade 3–4 AEs=18 (26%)	SABR was considered safe, and the irradiated lesions responded well
***SABR for Oligoprogressive RCC in Combination with Systemic Therapy***						
Cheung et al. (2021), single-arm, mRCC with ≤5 progressing sites on TKI [29]	TKI therapy followed by 3-8 fractions (total dose 48–60 Gy) or 3–6 fractions (total dose 30–60 Gy) or 5 fractions, (total dose 30–40 Gy), 1–5 fractions (total dose18–40 Gy), or 1–5 fractions (total dose 15–30 Gy) SABR	37	11.8	93% LC. Median time from SABR to the onset of new systemic therapy = 12.6 months	No grade ≥3 toxicity	Administering SABR to progressing sites during oral targeted therapy can significantly delay the need to transition to an alternative treatment regime
Hannan et al. (2022) single-arm mRCC with ≤3 progressing sites [31]	ICI/TKI/ICI+TKI plus One fraction (total dose ≥25 Gy) or three fractions (total dose ≥12 Gy), or five fractions (total dose ≥8 Gy)	20	10.4	100% LC. Median time from SABR to the onset of new systemic therapy = 11.1 months Median duration of SABR-aided systemic therapy = 24.4 months	1 patient developed grade 3 toxicity	The duration of the ongoing systemic therapy was boosted by the safe and efficient application of SABR to progressive locations
***SABR to Defer Systemic Therapy in Oligometastatic Renal Cell Carcinoma Patients***						
Tang et al. (2021), single-arm, mRCC ≤5 lesions with [32]	1 prior systemic therapy Ten fractions (Total dose 60–70 Gy) or 15 fractions (Total dose 52•5-67•5 Gy) SABR. All patients had a prior nephrectomy	30	18	97% LC Median PFS:23 months	3 grade 3/4 adverse events	Delaying the commencement of systemic therapy may be aided by SABR
Hannan et al. (2022) single-arm, mRCC ≤3 lesions [33]	No prior systemic therapy. One fraction (total dose 25 Gy) or three fractions (total dose 12 Gy) or five fractions (total dose 8 Gy) SABR	23	21.7	100% LC. Freedom fromsystemic therapy at 1yr=91%	1 grade ≥3 AEs (death from immune-related colitis)	The initiation of systemic therapy can be safely delayed with SABR
***SABR Deferring Cytoreductive Nephrectomy Combined with Immunotherapy in mRCC***						
Lalani et al. (2023), randomized, multi-center [34]	Untreated mRCC i. Arm A: I/N plus SABR (30–40 Gy in 5 fractions) to the primary kidney ii. Arm B: I/N alone	78	12	PFS, OS and quality of life	**-**	**-**
Hall et al. (2023) [35]	i. Arm A: I/N plus SABR (42 Gy in three fractions) to the primary kidney (n=160) ii. Arm B: I/N alone (n=80)	240	24	Radiographic progression-free survival (rPFS) with progression as per iRECIST	-	-
***Dual ICI with SABR***						
Hammers et al. (2020), single-arm [36]	50 Gy in 5 fractions between the first and the second dose of N/I	25	35	Objective Response Rate=56%. PR=14/25 patients Median PFS=8.2 months	Grade 3–4 AEs=36%	Dual ICIs with SABR was proven to be safe and acceptable
***SABR in Primary and Metastatic Renal Cell Carcinoma***						
Svedman et al. (2006) single-center [37]	Metastatic or inoperable primary RCC, [8 Gyx4, 10 Gy x4, 15 Gy x2 or 15 Gy x3] SABR. All patients with metastatic disease had undergone nephrectomy	30	52	LC=98% OS =32 months	Grade I–II =90%	For patients with limited number of metastases, SABR may provide a therapeutic alternative to surgery

Abbreviations: SABR, Stereotactic ablative radiation therapy; ORR, overall response rate; RECIST, Response assessment Criteria in Solid Tumors; mRCC, metastatic Renal cell carcinoma; LC, Local control; OS,Overall survival; PFS,Progression free survival; AE, adverse effects; HD IL2, High-Dose Interleukin-2;TKI, Tyrosine Kinase Inhibitor; RCC, Renal cell carcinoma; I/N, Ipilimumab and Nivolumab: DCR, Disease control rate.

### 1. SABR in Combination with Systemic Therapy

#### 1.1. SABR for Oligometastatic RCC in Combination with Systemic Therapy

A single-arm, open-label, prospective phase 2 trial of SABR at various sites was carried out by Hannan *et al*. with the objective to assess if the addition of SABR to high-dose IL2 (HD IL2) could raise the overall response rate along with tissue analyses in oligometastatic RCC with ≤6 lesions. The study included thirty individuals with clear cell mRCC who underwent prior nephrectomy between August 2013 and August 2017. A median of two metastases with a combined diameter of >1.5 cm (minimum gross target volume ≥2 cm^3^) were treated with SABR. At least one site remained unirradiated to measure the radiographic response for the primary endpoint. Potentially, this change is assumed to increase the presentation of tumour antigens, which can synergize with ICIs. Thirty-one oligometastases in total – bone (n=8), liver (n=2), and lung (n=21) – were exposed to radiation. Less than eighty-four hours preceded the onset of the first HD IL2 cycle after the last SABR fraction had been administered. One-fraction (21–27 Gy) or three-fraction (26.5–33 Gy) regimens were preferred for the largest treatable disease site and symptomatic areas needing palliative or preventive radiation. Following the treatment, imaging studies and physical examinations were scheduled every eight weeks for the first eight months, and every twelve weeks for the subsequent two years. *Adverse Events* (AE) were recorded by using the Common Terminology Criteria for Adverse Events, version 4.0 (CTCAE 4.0) [24]. The overall response rate included patients with partial response (PR) or complete response (CR). The World Health Organization (WHO)-provided, immune-related RECIST (iRECIST), and RECIST v 1.1 criteria were compared. According to RECIST v1.1, the best overall response was the best tumour response until the last follow-up or illness progression. Five patients were determined to be ineligible based on RECIST v1.1 since they did not have measurable non-irradiated illness at baseline. Of the 25 patients evaluated by using RECIST,16 percent (4 out of 25 patients) had an overall response rate, and 8 percent (2 out of 25 patients) had complete responses. The median *Overall Survival* (OS) was 37 months. Among the AEs, grade ≥3 toxicity was reported among 22 patients. Thus, adding SABR did not improve the response rate to HDIL2 in mRCC patients in this study. However, tissue analyses suggest a possible correlation between the frameshift mutation load and tumour immune infiltrates [25].

To evaluate *Immunotherapy* (IO) plus SABR in patients with oligometastatic RCC≤5 lesions, Siva *et al*. conducted a phase 2 single-arm multi-institutional trial between November 25, 2016, and April 11, 2019. The trial enrolled thirty-one patients with oligometastatic clear cell renal cell carcinoma (ccRCC) from two Australian centres. Following SABR treatment for every lesion with either one fraction (total dose 20 Gy) or ten fractions (total dose 30 Gy), each person received eight rounds of Pembrolizumab. Eighty-three oligometastases in total: adrenal (n=8), bone (n=11), lymph node (n=12), and lung (n=43) and soft tissue (n=9) were exposed to radiation. A median of three metastases treated with SABR for each patient constituted the dataset. With 13% of grade 3 toxicity, FFLP (*Freedom From Local Progression*), PFS (*Progression-Free Survival*), and OS at the 28-month follow-up were 92%,45%, and 74%, respectively. Hence, this strategy exhibits excellent LC, resilient responses to treated metastases, a manageable AE profile, and encouraging PFS, which warrants further investigation [26].

#### 1.2. SABR in Combination with Systemic Therapy for Unselected mRCC

The NIVES study, a Phase 2 single-arm trial, was conducted to evaluate whether combining Nivolumab with SABR enhances the objective response rate compared to Nivolumab alone. Sixty-nine immunotherapy naïve patients with histologically confirmed mRCC progressed after second and third-line treatment from July 2017 to March 2019 were enrolled. A non-clear cell histology was present in 17% (n=12) of the cases, and 77% previously had a nephrectomy. Based on the *International Metastatic Renal Cell Carcinoma Database Consortium* (IMDC) standards, the majority of the patients (74%) were classified as having an intermediate or poor risk [27]. As per RECIST 1.1 criteria, the patients required at least two detectable non-brain disease sites, with at least one eligible for SABR. Additionally, at least one measurable, non-irradiated lesion was necessary for response assessment. After the initial Nivolumab injection, seven days later, SABR was given in three fractions of 10 Gy to a single metastatic lesion (the lesion with the largest diameter). Lung lesion (37%), lymph node (12%), bone (10%), and liver (7.0%) lesions were among the irradiation lesions. Until progression or treatment termination, whichever happened first, the patients had radiographic assessments of their response every 12 weeks using either computed tomography (CT) or magnetic resonance imaging (MRI), using the RECIST 1.1 criteria. CTCAE 4.0 was used to evaluate safety [24]. The objective response rate and the *Disease Control Rate* (DCR) were 17% and 55%, respectively, after a follow-up of 26 months. Compared to non-irradiated metastases (12%), the objective response rate of irradiated lesions (29%) was higher. Additionally, the DCR of irradiation metastases was shown to be high (85%). 18 patients (26%) had grade 3–4 treatment-related AEs with a median PFS of 5.6 months and a median OS of 20 months. While the primary objective of improving the objective response rate with Nivolumab plus SABR compared with Nivolumab alone from 25% to 40% was not reached, the technique was deemed safe, and it has shown favourable response of the irradiation lesions in mRCC with the advancement of the disease [28].

#### 1.3. SABR for Oligoprogressive RCC in Combination with Systemic Therapy

Treatment recipients often develop resistance owing to intratumoral mutational heterogeneity. An alternative systemic therapy should be prescribed for these patients. Unfortunately, the limited success of treatment options can eventually leave patients with no viable options at all. Taking this into account, two phase 2 trials were carried out to determine whether SABR plus systemic therapy can extend the course of systemic therapy without compromising the *Quality of Life* (QOL). Cheung *et al*. initiated the first single-arm prospective multicenter phase 2 research to assess the effectiveness of SABR in treating oligoprogressive RCC patients with ≤5 progressing sites on TKI therapy. The qualifying criteria for eligibility are the *International Metastatic Renal Cell Carcinoma Database Consortium* (IMDC) favourable or intermediate-risk group, histologically confirmed metastatic clear cell renal cell carcinoma and a prior stable response following three months of TKI therapy. All patients were on Sunitinib, except two who were on Pazopanib. From July 2014 to May 2019, 37 mRCC patients were recruited. According to RECIST 1.1, all advancing lesions must be complained to SABR. Following the SABR treatment for 57 oligoprogressive tumors in total, of which: Lung/pleural (n=21), Nonspine bone (n=12), Lymph nodes (n=7), Adrenal (n=4), Liver (n=4), Spine (n=3), Brain (n=3), Spleen (n=2), Pancreas (n=1), the same TKI therapy was maintained. The study protocol included diverse SABR doses based on the anatomical site: Lung/pleural: 3–8 fractions (total dose 48–60 Gy) or Liver: 3–6 fractions (total dose 30–60 Gy), or Adrenal/kidney/lymphadenopathy/nonspine bone: 5 fractions (total dose 30–40 Gy ), spine: 1–5 fractions (total dose 18–40 Gy ) and brain: 1–5 fractions (total dose 15–30 Gy). The patients were monitored every three months after the completion of SABR until the systemic therapy plan was modified in response to the tumour progression. Diagnostic body imaging, such as CT and MRI, was performed at every follow-up appointment. During the one-year follow-up, 93% of the irradiation tumours had LC; the OS was 92%, the median PFS was 9.3 months, and no grade ≥ 3 toxicity events according to the CTCAE version 4 were observed. The systemic TKI drug needed to be changed after 12.6 months. Thus, in patients with metastatic kidney cancer receiving oral targeted therapy, stereotactic radiotherapy can significantly delay the need to switch to another line of treatment when tumours show limited progression. Despite its early closure, this trial is the biggest prospective multicenter assessment of SABR utilization in oligoprogression metastatic cancer. The introduction of immunotherapy, which replaced TKI therapy alone, and the lack of multisite SABR capability are two factors that most likely led to the sluggish patient accrual into the trial [29].

Hannan *et al*. conducted another single-arm, phase 2 trial at a university medical centre and Parkland Health and Hospital System that enrolled 20 oligoprogressive RCC patients. Pathologically confirmed mRCC (of any histology) with IMDC favourable- or intermediate-risk disease were recruited between February 2019 and October 2020. Thirty-seven lesions with a median size of 3.4 cm were given SABR treatment. Liver (16.2%), non-spine bone (16.2%), and lung (27%) were among the most common treated sites. The essential criteria included at least one set of radiographic images showing the overall disease control while on the first to fourth line of systemic therapy with ≤3 Progressing sites. Each patient received either of the following the SABR doses: one fraction (total dose ≥25 Gy), three fractions (total dose ≥12 Gy), or five fractions (total dose ≥8 Gy) along with the systemic therapy. Eight patients received ICI, eight received TKI, and four received combination therapy [ICI + TKI, three patients; TKI + mammalian target of rapamycin (mTOR-I), one patient]. Systemic therapy was optionally held from 3 days before to 3 days after SABR. CTCAE v 5.0 scale was used to evaluate the toxicities [30]. At a median follow-up of 10.4 months, the trial demonstrated 100% local control, 1 grade 3 adverse event and the median turn-around time from SABR to the onset of a new systemic therapy or death was 11.1 months. SABR also extended the duration of the ongoing systemic therapy by >6 months in 14 patients with no discernible decrease in QOL. The study demonstrates that SABR for oligoprogressive mRCC was safe and effective, and that it has likewise increased the duration of the ongoing systemic therapy without compromising QOL [31].

### 2. SABR to Defer Systemic Therapy

Two prospective phase 2 single-arm trials have tested sequential SABR in patients with oligometastatic RCC without systemic treatment. At the MD Anderson Cancer Center, Tang and colleagues performed a single-arm, phase 2 trial with 30 oligometastatic RCC patients with clear cell histology who had at least five lesions each. The patients were enrolled between July 13, 2018, and September 18, 2020. Before being enrolled, all patients had nephrectomy, and none had received more than one previous systemic therapy. In cases where the lesion was susceptible to SABR, hypofractionated intensity-modulated radiation regimens of 60–70 Gy in ten fractions or 52.5–67.5 Gy in fifteen fractions were employed. Three (10%) patients experienced serious adverse effects during a median follow-up of 17.5 months: two grade 3 (back pain and muscular weakness), and one grade 4 (hyperglycemia). There was also 97% LC and a median PFS of 22.7 months. Sequential radiation therapy thus makes it feasible for some patients with oligometastatic RCC to delay the initiation of systemic therapy [32].

Hannan *et al*. enrolled 23 pathologically confirmed oligometastatic systemic therapy – naïve RCC patients of favourable or intermediate IMDC risk group for a single-arm phase 2 trial who had three or fewer lesions. All of these patients were candidates for SABR. Based on the proximity of organs at risk of toxicity, the dose/fractionation schedule consisted of one fraction (total dose 25 Gy), three fractions (total dose 12 Gy), or five fractions (total dose 8 Gy) to the periphery of the target to cover >95% of the target volume. At a median follow-up of 21.7 months, LC was 100%. Freedom from systemic therapy at 1 year was 91.3%, with 82.6% one-year PFS. As per the CTCAE 5.0 scale, only one grade 3/4 side effect was detected, and there was no significant change in QOL from the baseline. Thus, SABR alone for oligometastatic RCC was associated with meaningful longitudinal disease control while preserving QOL [33].

### 3. Primary Site SABR with Systemic Therapy

In lieu of cytoreductive nephrectomy, two ongoing trials aimed to assess the use of IO in conjunction with testing SABR to the primary kidney tumour in patients with mRCC were conducted. The phase 2 randomized clinical study CYTOSHRINK (NCT04090710) is conducted in Canada and Australia with an accrual goal of 78 biopsy-proven mRCC (any histology) with IMDC intermediate/poor risk disease and a lesion of <20cm for patients who have not received therapy previously. Patients with primary tumours are treatable with SABR; they receive a 2:1 randomization to receive 30 to 40 Gy of SABR in five fractions plus Ipilimumab and Nivolumab, or Ipilimumab and Nivolumab as IO [34]. The primary variables used to compare the two arms are PFS, safety assessment, OS, the objective response rate, and QOL. Another phase 2 randomized trial, SAMURAI (NCT05327686), is being conducted as part of the NRG GU-012 clinical trial. It is intended for people with mRCC and is recruiting patients through the NRG oncology cooperative group to enrol 240 patients. Patients in this trial are randomized at a rate of 2:1 to receive 42 Gy of SABR in three fractions to primary plus Ipilimumab and Nivolumab versus Ipilimumab and Nivolumab. The goal is to compare the two arms based on PFS as per iRECIST [35].

### 4. Dual ICI with SABR

At UT Southwestern and Johns Hopkins, another multi-institutional, single-arm phase 2 study was undertaken to evaluate the safety and effectiveness of SABR (up to two metastatic locations) in combination with Nivolumab and Ipilimumab. After screening twenty-nine patients with clear cell mRCC, 25 were included with a life expectancy of at least three months and more than six months following the last dose of Neoadjuvant therapy that included TKI and IL2. The cohort of patients mainly included favourable risk = 2 (8%), intermediate risk = 20 (80%), and poor risk = 3 (12%) based on IMDC. The standard of care for the enrolled patients involved three weekly doses of Ipilimumab and Nivolumab, followed by Nivolumab monotherapy. Between the first and the second doses of Nivolumab and Ipilimumab, the disease sites (n=1,2) received 50 Gy of SABR divided into five fractions. At 35 months follow-up, the primary objective of this exploratory investigation was to determine the objective response rate, which was found to equal 56%. PR was noted in 14 out of 25 patients, with a median PFS of 8.2 months. Additionally, according to CTCAE v 4.0, 36% of the patients had toxicity grade 3–4 recorded. Dual ICI with SABR demonstrated a satisfactory level of safety and a promising antitumor efficacy in mRCC, thereby indicating the need for more research [36]

### 5. SABR in Primary and Metastatic Renal Cell Carcinoma

An open, single-centre phase 2 randomized prospective clinical research by Svedman *et al*. to evaluate the safety and local efficacy of high-dose fraction SABR in patients with metastatic or incurable primary renal cancer was executed from April 1999 to September 2004. The study involved thirty participants. The trial was open to patients with more than three months of life with inoperable primary, local recurrence, or mRCC. All of the individuals with metastatic illness had undergone nephrectomies. In total, eighty-two lesions with a minimum size of 10 mm were addressed. The dose/fractionation schedules were influenced by the target’s size and location (8 Gyx4, 10 Gyx4, 15 Gyx2 or 15 Gyx3). 98% of the treated patients had achieved LC, defined as partial/complete response (PR/CR) or radiologically stable disease (SD), at a median follow-up of 52 months. The OS was 32 months, and grade I–II AEs were present in 90% of cases. Thus, it is observed that in individuals with primary and metastatic RCC, SABR produced a high rate of LC with relatively few side effects [37].

## Mechanism of Action

Based on experimental and clinical research, radiation therapy (RT) may function as an *in-situ* cancer vaccine by immune-stimulating cell death, which releases antigens from the tumour and attracts CD8+ T cells to the location, increasing the tumour’s immunogenicity. Consequently, this may set off an immune reaction that is potentially anti-neoplastic, leading to tumour responses outside the areas exposed to radiation [38,39]. As previously reported in various cancer types [40–42], this phenomenon is known as the ‘abscopal’ influence. RCC has historically been considered a subtype of radioresistant tumours [12]. A high expression of *Hypoxia-Inducible Factor 1 Alpha* (HIF1A), a transcription factor inhibiting endothelial cell death in the tumour microenvironment, is the resistance to conventionally fractionated radiation therapy [43,44]. Through the inhibition of HIF1A overexpression and the induction of an endothelial death wave, SABR can circumvent this radioresistant mechanism [45,46].

On the other hand, the superior LC rates shown in RCC treated with SABR are explained by the initiation of this apoptotic signalling cascade [47]. Additionally, in radiobiologic cell survival tests, RCC has been shown to be a low alpha/beta malignancy, making it more vulnerable to greater doses per fraction when administered with SABR [48]. According to preclinical research, the optimal dose of hypofractionated radiation therapy to generate effects on the systemic immune system is 3 × 8 Gy [49]. Thus, with this novel approach, it is possible to safely administer this extremely precise hypofractionated radiation therapy with minimal side effects. The ‘abscopal’ effect, which occurs when the radiation therapy combined with ICIs leads to favourable local responses in irradiated cancers, is supported by clinical data and systemic abscopal impact [39].

## Factors Influencing SABR Impact

According to the evidence provided here, SABR may play a role in palliation, improving systemic therapy, or postponing systemic therapy in oligometastatic RCC and delaying therapy switching following oligoprogression. Nevertheless, a phase 2 study found that SABR did not increase the response rate to HDIL2 in patients with mRCC receiving HD IL2 [25]. Meanwhile, their analysis of somatic mutations revealed that RCC patients with high levels of frameshift mutations may be more likely to respond to SABR/IL2. Further research is still necessary to ascertain whether a frameshift mutation load can be combined with other selection criteria, such as clear cell histology and a favourable prognostic grouping, to identify patients for HDIL2 therapy [50]. Additionally, based solely on limited retrospective data, making firm recommendations for IO+SABR is challenging. Fortunately, Siva *et al*.’s recently published prospective mRCC trials offer additional insight into combined SABR and IO, primarily with ICI with remarkable LC and controllable AE.

In contrast to the retrospective analysis, the RAPPORT trial’s selected mRCC cohort, which included clear cell histology, oligometastatic illness, no receipt of SABR for brain metastasis, and none of whom had denovo disease at the time of diagnosis, may be the reason for the better OS seen in these patients [26,51]. Comparable in structure, the NIVES trial assessed extracranial SABR after Nivolumab and showed that the combination modality therapy was well-tolerated; nevertheless, it did not discover an increase in the objective response rate with SABR vs. Nivolumab controls alone [28]. However, because patients in the NIVES study may only have been included if they had a broadly metastatic illness and non-clear cell histology, their risk was higher than that of the RAPPORT experiment. In addition, the experiment did not treat all metastatic sites; rather, it just required one extracranial site to be susceptible to SABR (30 Gy in 3 fractions). In the RAPPORT study, Pembrolizumab was initiated 2 to 8 days after SABR, whereas Nivolumab was started 7 days before SABR in the NIVES trial. It is crucial to investigate whether the timing of IO, before or after SABR, might impact OS and affect the prognosis of patients with mRCC.

LC of a dominating area of progression with SABR may postpone switching to substantially higher-risk next-line systemic therapy, as demonstrated in two recently published trials by Cheng *et al*. and Hannan *et al*. in oligoprogressive RCC, thus helping to maintain QOL [29,31]. The absence of grade 3/4 toxicities is another possible advantage of SABR, as opposed to the 46–83% of patients who experience grade 3 toxicity while using current first-line systemic treatments [4–8]. Although there is little experience, phase I trials, both prospective and retrospective, indicate that concurrent ICI with SABR is safe [52,53]. It is also possible for certain patients with oligometastatic RCC to postpone the start of systemic therapy by using sequential radiation therapy [32,33]. In both primary and metastatic RCC, SABR resulted in a high incidence of local control and few side effects [37]. In phase II SAbR-COMET, patients with metastatic cancer and five or fewer metastases were randomized to receive the standard therapy or the standard care, plus SABR and OS were better in the SABR arm [54]. Thus, the procedure can be considered a non-surgical therapeutic option for individuals with few metastases, a local treatment for slowly progressing RCC or a means of reducing the tumour burden before seeking medical care. While one phase 2 trial demonstrated an encouraging antitumour efficacy in mRCC and acceptable safety when dual ICI with SBRT was used, there was no difference in the median PFS of the CheckMate-214 trial in which clinically meaningful results with ICIs were observed in the first-line setting [36,55]. These findings imply that various multidrug combinations should be assessed for their potential to work synergistically with SABR, especially in patients with oligometastatic or oligoprogressive diseases. The outcomes of prospective studies on the benefits of combined modality treatments in mRCC will continue to be provided by CYTOSHRINK (NCT04090710) [34] and SAMURAI (NCT05327686) [35]; remarkably, the clinical behaviour of radiographic oligometastatic RCC varies; some patients may benefit from upfront SABR, while others with a more indolent illness may require merely monitoring; yet others with more aggressive disease biology may require systemic therapy. Further research should be carried out on patient selection, biologic/molecular classification, histology and disease presentation impact, optimal combination treatments, SABR dosage, fractionations, and sequencing, as there are currently no recognized prognostic indicators for oligometastatic RCC. However, despite the limitations of the evidence, including non-standard SABR dose/fractionation, small and selected cohorts, single-arm designs, brief follow-up times, and contemporary doublet treatments, this approach is being used in clinical settings.

## Treatment Recommendations

A relatively large number of small studies have investigated using SABR for mRCC. In 2019, the *National Cancer Care Network* (NCCN) guidelines [56] began incorporating stereotactic ablative radiation as a treatment option for recurrent and metastatic RCC. SABR is defined by the *American Society for Therapeutic Radiation Oncology* (ASTRO) [57] as a treatment which combines very high doses (usually >5–8 Gy per fraction; however, a specific dose is not listed) of extremely precise and accurate, externally generated, ionizing RT to maximize the cell-killing effect on the target(s) while minimizing radiation-related injury in the adjacent tissue. As an ablative treatment for intact extracranial metastases for oligometastatic mRCC in SABR-amenable patients, SABR may be explored for medically inoperable patients. It can also be offered as a palliative approach to symptomatic extracranial metastases with strict adherence to the normal tissue constraints recommended (category 2A), according to NCCN guidelines, Version 1.2025 [58].

## Conclusion

With promising clinical results for a subgroup of mRCC patients with oligometastases and oligoprogression instead of and in addition to the systemic therapy, in prospective clinical trials, SABR has emerged as a compelling therapeutic option in metastatic disease. There are also active clinical trials investigating the use of SABR in conjunction with IO and systemic treatments [34,35,59]. More phase 3 trials are needed to support SABR robustly in the NCCN guidelines. This update to the earlier international analysis offers important information about the safety, effectiveness, and potential future applications of the approach, supporting its use in this subset of mRCC patients.

## Data Availability

This is a review article, not an original article. No new data has been published. All the data has already been submitted in the manuscript and table, as presented above.
